# High-Temperature Tribological Behavior and Wear Mechanisms of Stellite 6 Alloy

**DOI:** 10.3390/ma19122629

**Published:** 2026-06-18

**Authors:** Kai Jiang, Hongbin Lu, Weijie Chen, Fei Sun, Zhe Luo, Xiaomeng Gu

**Affiliations:** 1 School of Computer Science and Software, Shenzhen University of Information Technology, Shenzhen 518172, China; jiangkai@suit-sz.edu.cn; 2 School of Materials Science and Engineering, Fujian University of Technology, Fuzhou 350118, China; lhb20011020@163.com (H.L.); j2353426513@163.com (W.C.); sunfei2016@fjut.edu.cn (F.S.); 3 College of Intelligent Manufacturing, Hunan University of Science and Engineering, Yongzhou 425199, China

**Keywords:** Stellite 6 alloy, high-temperature wear, wear mechanism, oxide tribofilm

## Abstract

The temperature-dependent wear behavior of a cobalt-based Stellite 6 alloy was investigated from room temperature (RT) to 800 °C using high-temperature reciprocating sliding tests. The friction coefficient decreases monotonically with increasing temperature, from about 0.56 ± 0.12 at RT to 0.26 ± 0.11 at 800 °C, whereas the wear rate exhibits a pronounced non-monotonic evolution. Specifically, the wear rate increases from 18.4 ± 1.5 × 10^−6^ mm^3^·N^−1^·m^−1^ at RT to a maximum of 54.8 ± 1.6 × 10^−6^ mm^3^·N^−1^·m^−1^ at 600 °C, followed by an anomalous reduction to 10.2 ± 1.5 × 10^−6^ mm^3^·N^−1^·m^−1^ at 800 °C, which is even lower than that at RT. Microstructural and elemental analyses indicate that this behavior is governed by the temperature-dependent evolution of oxide layers. At RT–600 °C, thin and mechanically unstable oxide films repeatedly form and fracture, promoting oxidation-assisted abrasive and adhesive wear. In contrast, at 800 °C, a continuous and dense oxide layer forms and acts as a stable tribo-oxide film, effectively suppressing severe material removal. These findings clarify the temperature-driven wear mechanism transition of Stellite 6 alloy under high-temperature sliding conditions.

## 1. Introduction

Cobalt-based Stellite alloys represent a class of hardfacing materials widely recognized for their exceptional resistance to wear, corrosion, and high-temperature degradation [1,2,3]. Among them, Stellite 6 is regarded as the most versatile and widely used grade due to its balanced combination of mechanical strength, thermal stability, and the ability to maintain hardness across a broad temperature window [4,5]. Its microstructure features a face-centered cubic Co-rich solid-solution matrix strengthened by Cr-rich carbides, resulting in a distinctive combination of toughness, resistance to abrasive wear, and stability under high-temperature conditions [6,7]. The presence of these hard carbides, together with the strong solid-solution strengthening effect, imparts excellent tribological performance even under conditions where severe mechanical interactions and oxidative media coexist [8,9]. These intrinsic advantages make Stellite 6 a preferred material choice for demanding applications where surfaces are subjected to elevated temperatures, fluctuating contact stresses, and chemically aggressive environments [10,11].

The increasing performance requirements of critical components in aerospace, petrochemical processing, thermal power equipment, and nuclear engineering have amplified the demand for reliable surface protection materials capable of extended service under coupled thermal–mechanical loading [12,13]. Components such as turbine blades, valve seats, pump internals, and control rod drive mechanism (CRDM) parts routinely operate under complex combinations of sliding contact, thermal gradients, erosion, and corrosive atmospheres [14,15,16]. Under such extreme service conditions, the surface chemistry, microstructure, and mechanical response of the material can be significantly altered, making high-temperature tribological stability a decisive factor governing component reliability and service life [17]. Although Stellite 6 has been widely adopted in these industries, most existing studies focus primarily on its room-temperature or moderately elevated-temperature wear performance. Systematic data on its tribological behavior under harsh thermal conditions remain limited.

Recent investigations indicate that both the wear rate and frictional behavior of Stellite 6 alloy are strongly influenced by temperature [18,19]. At lower temperatures, the material generally maintains a relatively low and stable wear rate due to its high hardness and strong carbide reinforcement. As the temperature rises into the intermediate regime, the wear rate tends to increase, often attributed to the initiation of oxidation-assisted wear and changes in the stability of carbides. Correspondingly, the dominant wear mechanisms are known to evolve from abrasive and mild adhesive wear at low temperatures, to oxidation-assisted wear in the intermediate range, and may eventually involve severe oxidative wear or delamination, depending on the stability of oxide layers formed at high temperatures [20]. However, despite these observations, the underlying correlations between the temperature-dependent wear regimes, the evolution of oxide films, the stability and cracking of carbides, and the subsurface microstructural transitions remain insufficiently clarified. This lack of systematic understanding limits both the optimal utilization of Stellite 6 and the accurate prediction of its performance in extreme industrial environments.

In recent years, the high-temperature tribological behavior of Stellite 6 and related Co-based systems has attracted increasing attention. Ostolaza et al. [21] investigated functionally graded Stellite 6/WC composite coatings at room temperature and 400 °C and reported a transition from abrasive to oxidative wear with increasing temperature. Félix-Martínez et al. [22] further evaluated Stellite-6/WC-6Co coatings and showed that carbides provide a lubricating effect at room temperature while tribo-oxidation forms protective oxide layers at high temperature. Studies on thermally sprayed Stellite 6 coatings have further shown that, at 800 °C, a protective “glaze” oxide tribofilm can form on the worn surface and markedly reduce the wear rate and friction coefficient [23]. For bulk Stellite 6, Gurram et al. [18] examined its temperature-dependent sliding wear and found that oxidation- and delamination-controlled mechanisms dominate at elevated temperature, with the counterpart material strongly influencing the formation of a (Cr, O)-rich glaze layer. Despite these advances, most investigations have focused on coatings produced by specific deposition routes or have examined only a limited temperature range or a single elevated temperature. Systematic studies that resolve the full non-monotonic evolution of the wear rate across a wide temperature window, and that connect this evolution to the stability of the oxide films, the cracking of carbides, and the subsurface microstructural response, remain scarce. This gap motivates the present systematic investigation of bulk Stellite 6 under reciprocating sliding from RT to 800 °C.

Therefore, a comprehensive investigation of the temperature-dependent sliding wear behavior of Stellite 6 across a wide temperature window is essential for advancing both fundamental tribological knowledge and engineering practice. Unlike most previous studies, which have largely addressed Stellite 6 coatings at a single elevated temperature or over a limited temperature range, the present work focuses on bulk Stellite 6 across a wide temperature window from RT to 800 °C. In this work, high-temperature reciprocating sliding wear tests were conducted from RT to 800 °C to simulate severe thermo-mechanical service conditions. The results reveal a monotonic decrease in friction coefficient with increasing temperature, whereas the wear rate exhibits a pronounced non-monotonic evolution, reaching its maximum at 600 °C and then decreasing anomalously at 800 °C to a level even lower than that at RT. By combining quantitative wear evaluation with SEM-based wear track observations, cross-sectional BSE characterization, and elemental analysis, this study links the temperature-driven wear response to the formation and mechanical stability of surface oxide layers as well as subsurface damage development. These findings clarify the dominant wear mechanisms across different temperature regimes and provide mechanistic insight for evaluating and applying Stellite 6 under extreme high-temperature sliding conditions.

## 2. Experimental

### 2.1. Sample Preparation

The Stellite 6 alloy utilized in the present investigation was procured from Kennametal Stellite Co., Ltd., Shanghai, China, a reputable supplier of commercial cobalt-based hardfacing materials. The material was sectioned into block-shaped specimens with dimensions of 20 mm × 20 mm × 5 mm using a low-speed wire electrical discharge machining (WEDM; AP250L, Sodick Co., Ltd., Yokohama, Japan) cutter in order to minimize thermal damage and preserve the intrinsic microstructure. Prior to the friction and wear tests, all specimens underwent a standardized surface preparation procedure. The sample surfaces were sequentially ground using SiC abrasive papers of progressively finer grit sizes, followed by mechanical polishing with diamond suspensions to achieve a smooth and uniform finish. The polished samples were then ultrasonically cleaned in ethanol to remove any surface contaminants, debris, or residual polishing media. The chemical composition of the Stellite 6 alloy used in this study is presented in Table 1.

### 2.2. Microstructure Characterizations

The field-emission scanning electron microscopy (FE-SEM; Quanta FEG 450, FEI Company, Hillsboro, OR, USA) was used to observe the surface morphology of the polished Stellite 6 specimens. Both secondary electron (SE) and backscattered electron (BSE) imaging modes were applied to record microstructural features. The energy-dispersive X-ray spectroscopy (EDS) system attached to the SEM was used to collect elemental distribution information from selected regions. The X-ray diffraction (XRD; MiniFlex 600, Rigaku Corporation, Tokyo, Japan) with Cu Kα radiation was used to record the diffraction patterns of the Stellite 6 specimens. Scans were conducted over a 2θ range of 20–90°. The confocal laser scanning microscope (CLSM; VK-X1000, Keyence Corporation, Osaka, Japan) was used to measure the three-dimensional morphology of the wear tracks after the friction tests. Surface height profiles were collected to determine wear track depth, width, and volume loss.

### 2.3. High-Temperature Wear Testing

**Figure 1:**
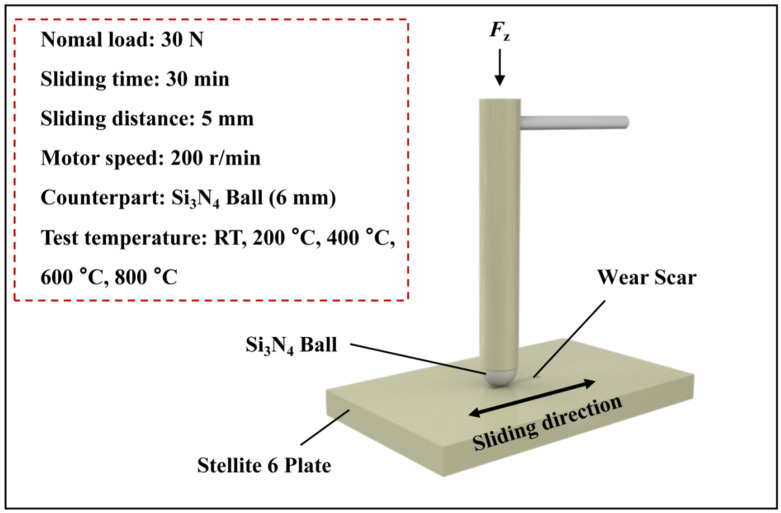
Schematic illustration of the high-temperature reciprocating wear test.

As shown in Figure 1, the high-temperature wear tests were performed using a GF-I high-temperature reciprocating friction and wear tester developed by Lanzhou Zhongke Kaihua Co., Ltd. (Lanzhou, China), to evaluate the friction and wear behavior of the Stellite 6 alloy at elevated temperatures. The reciprocating wear tests were carried out under a normal load of 30 N, with a stroke length corresponding to a 5 mm sliding distance in each direction. A silicon nitride (Si_3_N_4_) ball with a diameter of 6 mm was used as the counterface material for its high hardness, high-temperature strength, and good chemical and thermal stability. The motor driving the reciprocating motion operated at a speed of 200 r/min, and each test was performed for a total duration of 30 min, during which the friction coefficient was continuously recorded by the system’s data acquisition unit. The tests were conducted at five temperature conditions: room temperature (RT), 200 °C, 400 °C, 600 °C, and 800 °C. Prior to each test, the specimen and counterface were held at the target temperature for 10 min to ensure thermal stabilization. After the tests, the wear tracks on the Stellite 6 specimens and the wear scars on the Si_3_N_4_ balls were ultrasonically cleaned in ethanol. The three-dimensional profiles of the wear tracks were subsequently measured using the confocal laser scanning microscope described in Section 2.2 to obtain wear depth, wear width, and calculated wear volume. The specific wear rate (*ω*) was calculated based on the volume loss *V* (mm^3^), applied normal load *F* (N), and total sliding distance *L* (m), using the standard equation [24,25,26]. All friction and wear tests were repeated three times under each temperature condition, and the results are reported as mean values ± standard deviations to ensure measurement reliability.

(1) $$\omega =\frac{V_{loss}}{FL}$$

## 3. Results

### 3.1. Initial Microstructure and Phase Characterization

**Figure 2:**
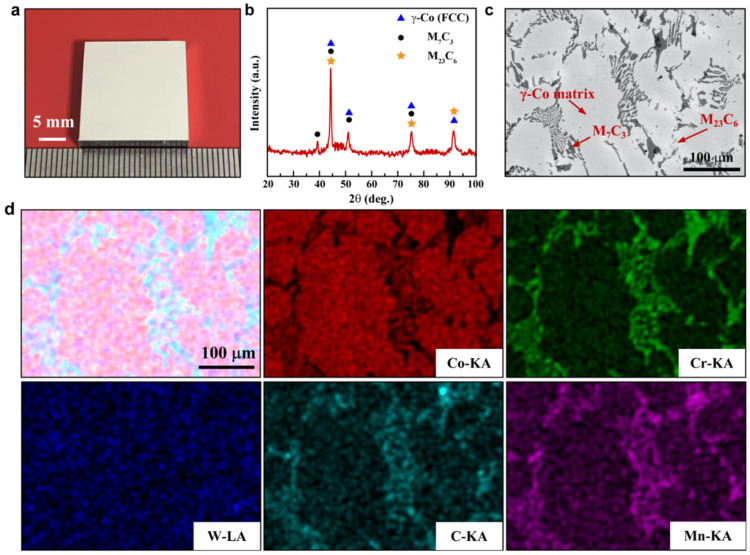
Characterization of the Stellite 6 alloy. (**a**) Optical surface morphology of the specimen before the wear test; (**b**) XRD pattern of the Stellite 6 alloy; (**c**) Microstructural observation under BSE mode; (**d**) Elemental distribution maps of the alloy.

Figure 2a shows the optical image of the Stellite 6 specimen prior to the high-temperature wear tests. After sequential grinding and polishing, the specimen exhibits a smooth and uniform surface with high surface finish, indicating that surface defects and machining-induced irregularities were effectively removed. This ensures good consistency and reliability for the subsequent high-temperature wear tests. Figure 2b presents the XRD pattern of the Stellite 6 alloy, in which the dominant diffraction peaks can be indexed to a Co-based solid-solution phase with an FCC structure. In addition, several weaker diffraction peaks can be indexed to carbide phases, including Cr-rich carbides such as M_7_C_3_ and/or M_23_C_6_, which is consistent with previous literature reports on Co–Cr–W–C hardfacing alloys [20,27]. Correspondingly, the microstructural features observed under the BSE mode in Figure 2c reveal a Co-rich matrix containing dispersed bright-contrast regions. Meanwhile, the EDS elemental maps shown in Figure 2d indicate that Cr and Mn are preferentially enriched in the bright-contrast regions, whereas Co is mainly distributed in the surrounding matrix. The localized enrichment of Cr and Mn suggests the presence of Cr- and Mn-rich carbides, which is in good agreement with the XRD results. This microstructural configuration, characterized by a Co-based matrix reinforced with dispersed carbide phases, provides the structural basis for the high-temperature wear behavior of the Stellite 6 alloy.

### 3.2. High-Temperature Wear Behavior at RT-400 °C

**Figure 3:**
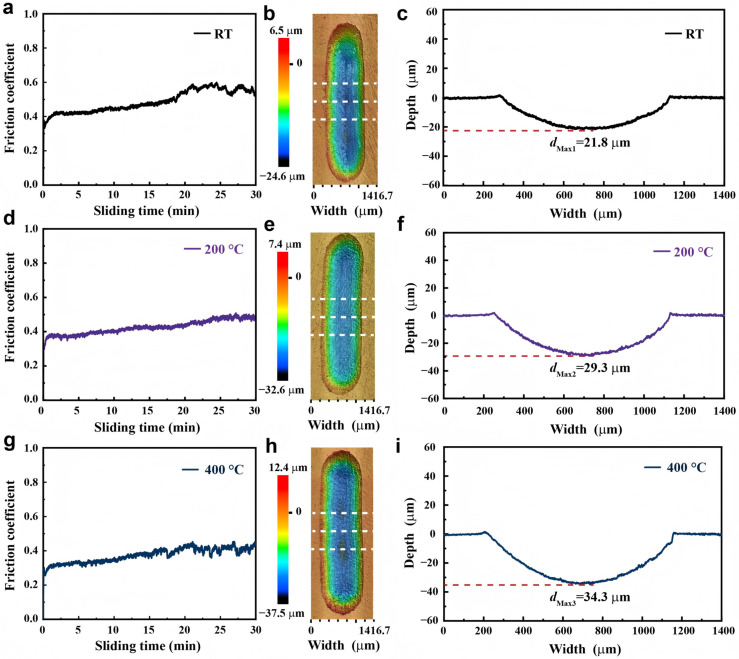
High-temperature wear behavior of the Stellite 6 alloy at different temperatures. (**a**) Friction coefficient curve at RT; (**b**) 3D morphology of the wear track at RT; (**c**) Averaged wear profile at RT; (**d**) Friction coefficient curve at 200 °C; (**e**) 3D morphology of the wear track at 200 °C; (**f**) Averaged wear profile at 200 °C; (**g**) Friction coefficient curve at 400 °C; (**h**) 3D morphology of the wear track at 400 °C; (**i**) Averaged wear profile at 400 °C. In the 3D morphology images (**b**,**e**,**h**), the white dashed lines indicate the equally spaced positions used for cross-sectional sampling to obtain the averaged wear profile.

Figure 3a shows the friction coefficient curve of the Stellite 6 alloy at RT. After a short running-in stage, the friction curve gradually enters a steady-state wear regime. The average friction coefficient is approximately 0.56, while pronounced fluctuations are observed during sliding. Such fluctuations can be attributed to the heterogeneous microstructure of the Stellite 6 alloy, in which a Co-rich matrix is reinforced by hard carbide phases [28,29]. During sliding at RT, intermittent interactions between the hard carbides and the counterface may cause local micro-cutting, carbide pull-out, or transient adhesive contacts, leading to unstable contact conditions and resulting in fluctuations in the friction coefficient. Subsequently, Figure 3b presents the 3D morphology of the wear track formed at RT. Wear profiles were extracted at intervals of 200 μm across the wear track, and the averaged wear profile is shown in Figure 3c. The maximum wear depth (d_1_) is measured to be 21.8 μm. Based on the CLSM analysis, the wear volume at RT is determined to be 33.2 × 10^−3^ mm^3^. Accordingly, the wear rate calculated using Equation (1) is 18.4 ± 1.5 × 10^−6^ mm^3^ N^−1^ m^−1^. In comparison, Figure 3d shows the friction coefficient curve of the Stellite 6 alloy at 200 °C. Compared with RT, the average friction coefficient decreases to approximately 0.41, and the friction curve becomes relatively smoother. This behavior can be associated with the formation of a thin oxide layer on the sliding surface at elevated temperature, which may partially separate direct contact at the interface and contribute to a more stable friction response. Correspondingly, Figure 3e,f show the 3D morphology and the averaged wear profile of the wear track formed at 200 °C, respectively. The maximum wear depth increases to approximately 29.3 μm, and the corresponding wear volume is 53.0 × 10^−3^ mm^3^. The wear rate at 200 °C is therefore calculated to be 29.4 ± 2.0 × 10^−6^ mm^3^ N^−1^ m^−1^, indicating a higher material loss compared with RT. With further increasing temperature, Figure 3g presents the friction coefficient curve obtained at 400 °C. A similar trend to that observed at 200 °C is maintained; however, the average friction coefficient further decreases to approximately 0.38. This reduction can be attributed to the enhanced formation and accumulation of oxide films at higher temperatures [30,31]. These oxide layers can act as a solid lubricating medium during sliding, reducing the shear resistance at the contact interface and leading to a lower friction coefficient. Accordingly, the corresponding 3D wear morphology and averaged wear profile at 400 °C are shown in Figure 3h,i, respectively. The maximum wear depth increases to approximately 34.3 μm, and the wear volume reaches 66.2 × 10^−3^ mm^3^, resulting in a wear rate of 36.8 ± 1.9 × 10^−6^ mm^3^ N^−1^ m^−1^. Overall, with increasing temperature from RT to 400 °C, the wear depth increases from 21.8 μm to 34.3 μm, and the wear rate increases from 18.4 ± 1.5 × 10^−6^ mm^3^ N^−1^ m^−1^ to 36.8 ± 1.9 × 10^−6^ mm^3^ N^−1^ m^−1^, indicating that elevated temperature significantly aggravates material wear. Although oxidation induced by increasing temperature may exert a certain lubricating effect, as reflected by the reduced friction coefficient, the concurrent thermal softening of the material leads to an overall increase in wear severity [32].

**Figure 4:**
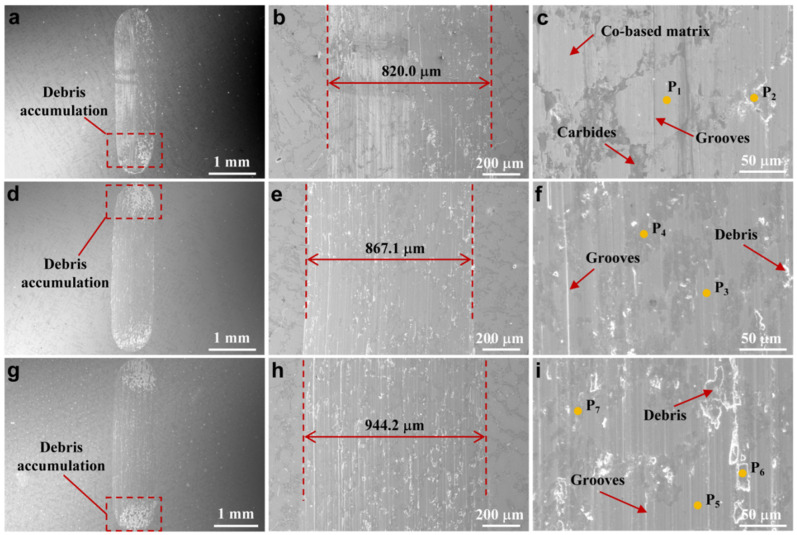
SEM morphologies of wear tracks on Stellite 6 alloy at different temperatures. (**a**) Low-magnification image at RT; (**b**,**c**) High-magnification images at RT; (**d**) Low-magnification image at 200 °C; (**e**,**f**) High-magnification images at 200 °C; (**g**) Low-magnification image at 400 °C; (**h**,**i**) High-magnification images at 400 °C.

To further clarify the wear behavior reflected by the friction coefficient and wear rate results in Figure 3, the surface morphologies of the wear tracks formed at RT, 200 °C, and 400 °C were examined by SEM, as shown in Figure 4. At RT, the low-magnification image in Figure 4a reveals limited wear debris accumulation at both ends of the wear track, resulting from repeated extrusion of debris during reciprocating sliding. The high-magnification images in Figure 4b,c show a wear track width of approximately 820.0 μm. The worn surface retains microstructural features similar to those of the as-prepared alloy, including the Co-based matrix and carbide phases, indicating that no pronounced surface oxidation occurs at RT. Shallow grooves are observed along the sliding direction, suggesting mild abrasive wear. Elemental analysis, summarized in Table 2, shows a low oxygen content of 4.40 at.% in the freshly exposed layer (P_1_), whereas the adhered debris region (P_2_) exhibits a much higher oxygen content (61.32 at.%), indicating that the debris mainly consists of oxidized particles. At 200 °C, wear debris accumulation at both ends of the wear track becomes more pronounced (Figure 4d). The wear track width increases to approximately 867.1 μm, as shown in Figure 4e,f, which is consistent with the increased wear volume in Figure 3. Grooves are still present on the worn surface, indicating the persistence of abrasive wear. The oxygen content in the freshly exposed layer (P_3_) remains low (4.14 at.%), while the gray region (P_4_) shows an elevated oxygen content (55.67 at.%). Combined with the reduced friction coefficient at this temperature, these results suggest that oxidation begins to participate in the wear process through a repeated oxide film formation–fracture–reformation mechanism. At 400 °C, as shown in Figure 4g, wear debris accumulation further increases. The wear track width, as measured in Figure 4h,i, reaches approximately 944.2 μm, in agreement with the further increase in wear volume. The density of grooves on the worn surface becomes higher, indicating intensified abrasive wear. Elemental analysis reveals increased oxygen contents of 10.54 at.%, 59.41 at.%, and 62.59 at.% in the freshly exposed layer (P_5_), adhered debris (P_6_), and gray oxidized region (P_7_), respectively.

### 3.3. High-Temperature Wear Behavior at 600–800 °C

**Figure 5:**
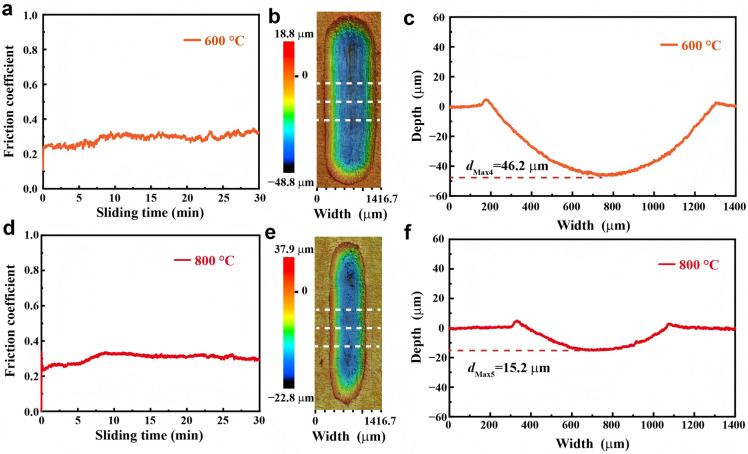
High-temperature wear behavior of the Stellite 6 alloy at 600 °C and 800 °C. (**a**) Friction coefficient curve at 600 °C; (**b**) 3D morphology of the wear track at 600 °C; (**c**) Averaged wear profile at 600 °C; (**d**) Friction coefficient curve at 800 °C; (**e**) 3D morphology of the wear track at 800 °C; (**f**) Averaged wear profile at 800 °C. In the 3D morphology images (**b**,**e**), the white dashed lines indicate the equally spaced positions used for cross-sectional sampling to obtain the averaged wear profile.

Following the wear behavior observed at lower temperatures, Figure 5a shows the friction coefficient curve of the Stellite 6 alloy at 600 °C. It can be seen that the average friction coefficient further decreases to approximately 0.29, which is consistent with the trend observed at 400 °C. This behavior indicates that the lubricating effect associated with oxide film formation becomes more pronounced with increasing temperature, leading to a further reduction in friction. Correspondingly, Figure 5b,c present the 3D morphology and the averaged wear profile of the wear track formed at 600 °C, respectively. The maximum wear depth is measured to be approximately 46.2 μm. Based on CLSM analysis, the wear volume at 600 °C is determined to be 98.7 × 10^−3^ mm^3^, resulting in a wear rate of 54.8 ± 1.6 × 10^−6^ mm^3^ N^−1^ m^−1^. Compared with the results obtained at lower temperatures, a pronounced increase in material loss is observed at 600 °C, indicating a deterioration in wear resistance at this temperature. With further increasing temperature, Figure 5d shows the friction coefficient curve obtained at 800 °C. A similar overall trend to that at 600 °C is maintained, while the average friction coefficient further decreases to approximately 0.26. The relatively low and stable friction coefficient suggests that the sliding interface is strongly influenced by high-temperature oxidation, which promotes sustained lubrication during the wear process. More importantly, Figure 5e,f show the 3D wear morphology and the averaged wear profile at 800 °C, respectively. In contrast to the severe wear observed at 600 °C, the maximum wear depth at 800 °C is significantly reduced to approximately 15.2 μm. The corresponding wear volume is 18.3 × 10^−3^ mm^3^, resulting in a wear rate of 10.2 ± 1.5 × 10^−6^ mm^3^ N^−1^ m^−1^. Notably, this wear rate is not only markedly lower than that obtained at 600 °C, but also lower than the wear rate measured at RT. Overall, as the temperature increases to 600 °C, the Stellite 6 alloy exhibits the most severe material loss. However, when the temperature is further increased to 800 °C, an anomalous reduction in wear rate is observed, suggesting that the dominant wear mechanism undergoes a significant transition at high temperature. This phenomenon will be discussed in detail in the subsequent section.

**Figure 6:**
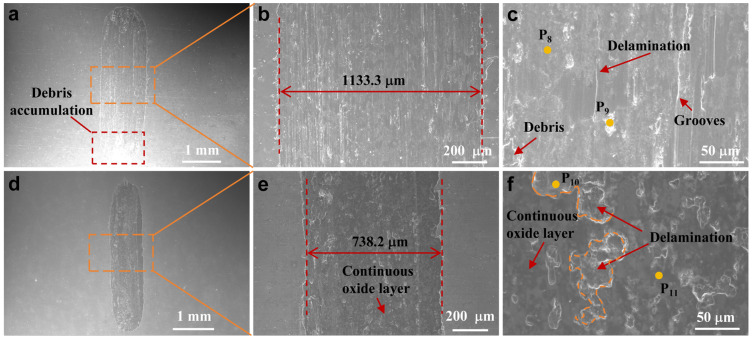
SEM morphologies of wear tracks on Stellite 6 alloy at different temperatures. (**a**) Low-magnification image at 600 °C; (**b**,**c**) High-magnification images at 600 °C; (**d**) Low-magnification image at 800 °C; (**e**,**f**) High-magnification images at 800 °C.

To further elucidate the wear behavior at elevated temperatures reflected in Figure 5, the wear track morphologies of the Stellite 6 alloy at 600 °C and 800 °C were examined by SEM, as shown in Figure 6. At 600 °C, the low-magnification image in Figure 6a reveals a significantly widened wear track. As shown in the high-magnification images in Figure 6b,c, the wear track width increases to approximately 1133.3 μm, indicating a pronounced increase in material removal, which is consistent with the increased wear rate observed in Figure 5. Numerous deep and continuous grooves are clearly visible on the worn surface, suggesting that abrasive wear is still operative at this temperature. In addition, adhered wear debris as well as delamination and spallation of surface oxide layers can be observed, indicating the simultaneous occurrence of adhesive wear and fatigue-induced delamination. Elemental analyses were performed on the freshly exposed layer (P_8_) and the bright oxide-covered region (P_9_), with the results summarized in Table 3. Both regions exhibit high oxygen contents of 62.36 at.% and 65.78 at.%, respectively, demonstrating that severe oxidation occurs at 600 °C. Combined with the further reduction in friction coefficient shown in Figure 5a, these observations indicate that oxidative wear becomes the dominant wear mechanism at this temperature. At 800 °C, a markedly different wear morphology is observed. As shown in Figure 6d,e, the wear track width decreases significantly to approximately 738.2 μm, which is not only much smaller than that at 600 °C but also lower than the value measured at RT. This observation is consistent with the reduced wear volume and wear rate at 800 °C. High-magnification examination in Figure 6f shows that the worn surface is characterized by a continuous oxide layer accompanied by large-area delamination features, while typical abrasive grooves are rarely observed, indicating that abrasive wear is no longer the dominant mechanism. Elemental analyses conducted on the oxide layer region (P_10_) and the delaminated region (P_11_) reveal high oxygen contents of 64.09 at.% and 62.36 at.%, respectively, confirming the occurrence of rapid and intense oxidation at this temperature. These results suggest that wear at 800 °C is governed by a cyclic process involving rapid formation of a dense oxide layer, its fracture and delamination under alternating stress, and subsequent rapid re-formation at high temperature [32,33].

### 3.4. Temperature-Driven Wear Mechanism Evolution of Stellite 6 Alloy

**Figure 7:**
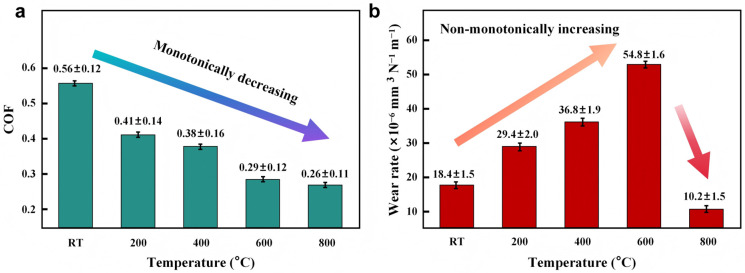
Statistical analysis of tribological behavior of Stellite 6 alloy at different temperatures. (**a**) The friction coefficient as a function of temperature; (**b**) The wear rate as a function of temperature.

Based on the results presented in the preceding sections, it can be clearly observed that the wear behavior of the Stellite 6 alloy is strongly governed by temperature, exhibiting a pronounced evolution of dominant wear mechanisms from RT to 800 °C. As summarized in Figure 7, the average friction coefficient decreases monotonically with increasing temperature, from 0.56 ± 0.12 at RT to about 0.26 ± 0.11 at 800 °C. In contrast, the wear rate exhibits a non-monotonic trend. Specifically, the wear rate increases from 18.4 ± 1.5 × 10^−6^ mm^3^ N^−1^ m^−1^ at RT to a maximum value of 54.8 ± 1.6 × 10^−6^ mm^3^ N^−1^ m^−1^ at 600 °C, and then decreases sharply to 10.2 ± 1.5 × 10^−6^ mm^3^ N^−1^ m^−1^ at 800 °C, which is even lower than that measured at RT. This distinct discrepancy between the monotonic reduction in friction coefficient and the non-monotonic evolution of wear rate indicates that friction alone cannot reliably reflect wear severity under elevated-temperature conditions. At RT, the relatively high friction coefficient combined with a moderate wear rate indicates unstable interfacial contact conditions. In this regime, wear is mainly governed by a combination of abrasive and adhesive mechanisms, originating from the heterogeneous microstructure of the alloy in which hard carbide phases intermittently interact with the counterface, leading to micro-cutting, local fracture, and adhesive junction formation. With increasing temperature to the intermediate range (200 to 600 °C), oxidation progressively participates in the wear process. Although the formation of surface oxides contributes to a reduction in the friction coefficient, the wear rate increases continuously and reaches its maximum at 600 °C, indicating aggravated material removal.

The contrasting trends in Figure 7 originate from different aspects of the surface oxide. The friction coefficient is primarily controlled by the presence of an oxide film at the sliding interface. As temperature increases, progressively more surface oxide forms and acts as a solid lubricant, continuously reducing the interfacial shear resistance, so that the friction coefficient decreases monotonically from approximately 0.56 at RT to 0.26 at 800 °C. The wear rate, however, is governed not by the mere presence of the oxide but by its mechanical stability and continuity. From RT to 600 °C, the oxide films are thin, discontinuous, and mechanically unstable, repeatedly forming and fracturing under reciprocating loading. Combined with thermal softening of the Co-based matrix and subsurface cracking along the carbide and matrix interfaces, this drives a continuous increase in wear rate that peaks at 600 °C. At 800 °C, the oxide becomes continuous, dense, and load-bearing, suppressing direct metal-to-counterpart contact and producing the anomalous reduction in wear rate. Because friction reflects whether a lubricating oxide is present, whereas wear reflects whether that oxide is stable and continuous enough to be protective, the two parameters become decoupled, with friction governed by the lubricating action of the oxide and wear governed by its load-bearing stability.

**Figure 8:**
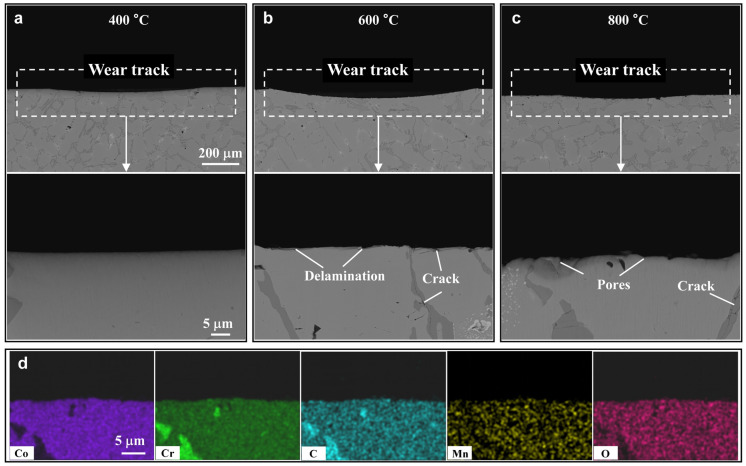
Cross-sectional characterization of wear tracks at different temperatures. (**a**–**c**) BSE images of wear track cross-sections at representative temperatures; (**d**) Corresponding EDS elemental distribution map showing oxygen enrichment near the wear surface at 800 °C.

To further elucidate the underlying wear mechanisms at different temperatures, cross-sectional analyses of the wear tracks were conducted. Figure 8a–c present BSE images of the wear track cross-sections formed at representative temperatures of 400 °C, 600 °C, and 800 °C, respectively. At all temperatures, the substrate beneath the wear surface retains a typical microstructural configuration consisting of a Co-based matrix reinforced by an interdendritic carbide network, indicating that no large-scale phase decomposition or bulk microstructural transformation occurs during high-temperature sliding. At 400 °C, as shown in the magnified image in Figure 8a, the Co-based matrix beneath the wear surface remains relatively intact. No pronounced cracks or pores are observed in the subsurface region, suggesting that fatigue-induced damage is not significant at this temperature. This observation is consistent with the moderate wear rate measured at 400 °C and indicates that material removal is mainly confined to the near-surface region without severe subsurface degradation. In contrast, at 600 °C, clear subsurface cracks and delamination features are observed, as shown in Figure 8b and its magnified view. These cracks preferentially propagate along the interfaces between the thermally softened Co-based matrix and the rigid interdendritic carbide network [32]. Such features suggest that matrix softening at high temperature, combined with the intrinsic brittleness of the carbide network, promotes crack initiation and propagation under cyclic shear stresses, thereby contributing to material loss through delamination. Moreover, distinct pores are observed near the wear surface at 800 °C, as shown in the enlarged image in Figure 8c. Combined with the elemental distribution map in Figure 8d, which reveals pronounced oxygen enrichment within these regions, these pores are attributed to the formation of a relatively thick and brittle oxide layer. Under alternating mechanical stress during reciprocating sliding, this oxide layer is prone to fatigue damage, leading to local cracking and delamination. Nevertheless, the continuous and hard oxide layer formed at 800 °C effectively suppresses severe material removal by reducing direct metal-to-counterface contact and bearing part of the applied load, thereby preventing catastrophic wear despite the presence of subsurface damage features. The continuous and dense Cr-rich oxide layer observed at 800 °C (Figure 8) also distinguishes the tribological behavior of Stellite 6 from that of Ni-based superalloys such as Inconel. In Ni-based superalloys, oxide scales formed under high-temperature sliding tend to be locally heterogeneous and mechanically unstable, appearing as discontinuous oxide islands rather than a continuous protective film; such local oxides promote abrasive wear by introducing hard debris into the contact zone instead of acting as a solid lubricant [34]. In the present Stellite 6 alloy, by contrast, the high Cr content facilitates the preferential formation of a dense and adherent Cr-rich oxide tribo-layer that remains continuous and stable under the applied reciprocating contact conditions, directly accounting for the anomalous wear reduction observed at 800 °C. This comparison highlights the critical role of oxide layer continuity and stability in determining the high-temperature wear performance of metallic alloys. Compared with conventional CoCr alloys and other Stellite grades, the high Cr content and carbide-reinforced cobalt matrix of Stellite 6 likewise favor the formation of a more stable and continuous protective oxide layer at elevated temperature, which underlies its competitive high-temperature wear resistance among these alloy systems.

**Figure 9:**
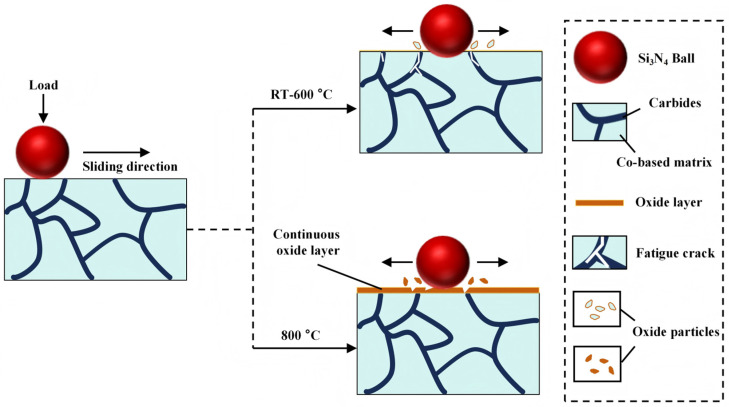
Schematic illustration of temperature-driven wear mechanism evolution of Stellite 6 alloy.

For clarity, the temperature-driven wear mechanisms of the Stellite 6 alloy are schematically illustrated in Figure 9. At RT–600 °C, the wear process is governed by a mixed mechanism involving abrasive wear, adhesive wear, and oxidation-assisted wear. Direct contact between the Co-based matrix, the interdendritic carbide network, and the counterface leads to micro-cutting and localized adhesion. With increasing temperature, thin and discontinuous oxide layers form on the sliding surface but remain mechanically unstable under reciprocating loading. These oxide layers undergo repeated formation, fracture, and removal, while thermal softening of the Co-based matrix promotes subsurface plastic deformation, crack initiation, and delamination along carbide–matrix interfaces, resulting in increased material removal toward the upper end of this temperature range. At 800 °C, the dominant wear mechanism changes markedly, as a continuous, dense, and hard oxide layer rapidly forms on the wear surface and acts as a stable tribo-oxide film. This oxide layer reduces direct metal-to-counterface contact and bears part of the applied load. Although subsurface cracking and local delamination may still occur due to matrix softening and the brittleness of the carbide network, the presence of this protective oxide layer effectively suppresses severe material removal, leading to a significantly reduced wear rate. This oxide layer-controlled wear reduction is governed primarily by the oxidation of the Stellite 6 substrate itself, rather than by the Si_3_N_4_ counterpart.

The formation of the protective oxide layer at 800 °C can be further understood in terms of oxidation kinetics, the Pilling–Bedworth ratio (PBR), and chromium enrichment. The thermally grown oxide is controlled by ionic diffusion and follows an approximately parabolic rate law, so its growth rate decreases as the scale thickens. At 600 °C, the oxidation rate is low and the thin film is repeatedly disrupted under reciprocating contact before a continuous protective scale can form, whereas at 800 °C, the markedly accelerated kinetics enable rapid re-formation and thickening, sustaining a continuous, self-replenishing layer even under mechanical removal. The protectiveness of this scale is consistent with the PBR of the preferentially formed Cr_2_O_3_ (≈2.0), which lies within the 1–2 range characteristic of dense, adherent oxides. Moreover, the high Cr content of Stellite 6 promotes preferential oxidation and surface enrichment of Cr, favoring a continuous Cr_2_O_3_-based scale over less protective Co-based oxides—consistent with the pronounced Cr-O co-enrichment detected by EDS and accounting for the stability of the tribo-oxide layer that governs the anomalous wear reduction at 800 °C.

It should be noted that the present study has several limitations. First, the mechanical response of the alloy at elevated temperature was inferred from the wear behavior and microstructural observations rather than from in situ high-temperature hardness measurements. Second, the wear tests were conducted under a single normal load and test duration; the influence of varying load and exposure time on the stability of the protective oxide layer was not systematically examined. Third, the present work focused on the temperature-dependent wear mechanism of the Stellite 6 specimen, and the Si_3_N_4_ counterpart was not separately characterized. These aspects, together with a more detailed phase-level characterization of the tribo-oxide layer, will be addressed in future work to further generalize the present findings.

## 4. Conclusions

Based on a systematic investigation of the high-temperature sliding wear behavior of Stellite 6 alloy from RT to 800 °C, the following conclusions can be drawn:

1. The tribological behavior of Stellite 6 alloy exhibits a strong temperature dependence from RT to 800 °C. While the friction coefficient decreases monotonically with increasing temperature, the wear rate shows a pronounced non-monotonic evolution, indicating that the friction coefficient alone is insufficient to assess wear severity at elevated temperatures.
2. As the temperature increases from RT to 600 °C, the wear rate rises significantly and reaches its maximum at 600 °C. This deterioration in wear resistance is associated with thermal softening of the Co-based matrix and the formation of thin, discontinuous, and mechanically unstable oxide layers that undergo repeated fracture and removal during sliding.
3. When the temperature is further increased to 800 °C, the wear rate decreases sharply to a level even lower than that measured at RT. This anomalous reduction is attributed to the formation of a continuous, dense, and hard oxide layer that acts as a stable tribo-oxide film, providing both lubrication and partial load-bearing capability.
4. The dominant wear mechanism of Stellite 6 alloy transitions from abrasion-adhesion-dominated wear at RT, to oxidation-assisted severe wear at intermediate temperatures, and finally to oxide layer-controlled wear at 800 °C, highlighting the critical role of oxide layer stability in governing high-temperature wear resistance.

## Figures and Tables

**Table 1:** Chemical composition of Stellite 6 alloy plate (wt.%).

Stellite 6 Alloy	Element Content (wt.%)						
	Co	Cr	W	Fe	C	Mo	Mn
	Bal.	28.80	4.37	2.16	1.02	0.20	0.16

**Table 2:** Elemental compositions (at.%) of selected regions on the wear tracks at RT-400 °C.

Point No.	Element Content (at.%)					
	Co	Cr	C	Mn	O	Others
P_1_	51.45	21.22	10.64	1.30	4.40	Bal.
P_2_	19.16	10.30	2.81	0.57	61.32	Bal.
P_3_	48.24	21.29	12.30	1.49	4.14	Bal.
P_4_	21.25	11.76	1.92	0.80	55.67	Bal.
P_5_	46.77	17.66	11.56	1.37	10.54	Bal.
P_6_	19.56	10.35	4.41	0.70	59.41	Bal.
P_7_	18.40	9.80	3.10	0.70	62.59	Bal.

**Table 3:** Elemental compositions (at.%) of selected regions on the wear tracks at 600–800 °C.

Point No.	Element Content (at.%)					
	Co	Cr	C	Mn	O	Others
P_8_	17.77	10.62	2.11	0.72	62.36	Bal.
P_9_	17.78	9.00	2.23	0.55	65.78	Bal.
P_10_	17.92	9.84	1.54	0.66	64.09	Bal.
P_11_	22.96	7.05	2.07	0.47	62.36	Bal.

## Data Availability

The original contributions presented in this study are included in the article. Further inquiries can be directed to the corresponding authors.
